# Atrofia Muscular Espinal: La Realidad del Paciente Adulto en España

**DOI:** 10.31083/RN33462

**Published:** 2025-03-27

**Authors:** Maria Grazia Cattinari, Mencía De Lemus, Maria Dumont, Eduardo Tizzano

**Affiliations:** ^1^Fundación de Atrofia Muscular Espinal, FundAME, 28034 Madrid, España; ^2^SMA Europe, 79112 Freiburg, Germany; ^3^Committee of Advanced Therapies at the European Medicines Agency, 1083 HS Amsterdam, The Netherlands; ^4^Medical Genetics Group, Rare Diseases Unit, Department of Clinical and Molecular Genetics, Vall d'Hebron Research Institute, Hospital Valle Hebron, 08035 Barcelona, España; ^5^CIBERER, Barcelona, España

**Keywords:** atrofia muscular espinal, enfermedades neuromusculares, registro de pacientes, enfermedad rara, datos de vida real, acceso a tratamiento, paciente adulto, RegistrAME, registro auto informado, spinal muscular atrophy, neuromuscular disorders, patient registry, rare disease, real-life outcome data, treatment access, adult patient, RegistrAME, self-reported registry

## Abstract

**Introducción:**

La atrofia muscular espinal (AME) es una enfermedad neurodegenerativa que durante la última década ha experimentado un aumento de la supervivencia gracias a un manejo multidisciplinar más temprano y proactivo, y a la aparición de terapias modificadoras de la enfermedad. A pesar de ello, siguen quedando un número importante de necesidades que impactan muy significativamente en la calidad de vida de las personas con AME. Ante la necesidad de obtener un mejor conocimiento de la patología, Fundación de Atrofia Muscular Espinal (FundAME) creó el estudio longitudinal (RegistrAME).

**Métodos:**

RegistrAME es un estudio prospectivo que recoge datos clínicos y variables referidas por los propios pacientes.

**Resultados:**

Se incluyeron 336 sujetos, el 51,8% tenían 16 años o más. La mayoría de la subpoblación adulta eran AME tipo 2 (49,4%) y tipo 3 (44,8%) y, atendiendo al nivel motor, el 19% caminaban (de los cuales el 39,4% usaba silla de ruedas), el 46,6% tenían sedestación sin apoyo (84% necesitaban ayuda de otra persona para conseguir la posición) y el 34,5% no lograban mantenerse sentados sin apoyo. El 24,7% reportaron no tener función útil en las manos o no poder alcanzar la boca con ellas. El estudio muestra un incremento progresivo del acceso, aunque un 21,8% de adultos no recibe tratamiento modificador de enfermedad.

**Conclusiones:**

Además del retraso o menor acceso a los tratamientos y ensayos, estos resultados evidencian deterioro progresivo y mayor susceptibilidad a discontinuaciones en adultos con AME. Se requiere un seguimiento más preciso del impacto de la enfermedad y del beneficio de los tratamientos recibidos.

## Abstract (en)

**Background:**

Spinal muscular atrophy (SMA) is a neurodegenerative disease in which there has been an increase in survival over the past decade due to proactive, multidisciplinary management and the emergence of disease-modifying therapies. Despite this, there are still several critical challenges that significantly impact the quality of life of individuals with SMA individuals. Given the need to better understand the reality of this disease, Fundación de Atrofia Muscular Espinal (FundAME) created a patient registry (RegistrAME).

**Methods:**

RegistrAME is a longitudinal prospective study that collects clinical data and patient-reported outcomes.

**Results:**

The registry included 336 individuals, 51.8% of whom were over 16 years old. Most adult subpopulations were classed as type 2 (49.4%) or type 3 (44.8%). Regarding functional status, 19% walked (39.4% used wheelchairs), 46.6% sat without support (84% needed help to sit), and 34.5% were non-sitters. Furthermore, 24.7% reported having no useful function in their hands or not being able to reach their mouths with their hands. Our study indicates that the adult SMA population is progressively accessing disease-modifier treatments. However, 21.8% of the population is still untreated.

**Conclusions:**

These results provide evidence of a progressive impairment and increased sensitivity to treatment discontinuations in the SMA adult population, along with delayed or reduced access to commercialised SMA drugs and clinical trials. A more rigorous evaluation of the disease’s impact and treatment benefits in the adult SMA population is necessary.

## 1. Introducción u Objetivos

La atrofia muscular espinal incluye un grupo de enfermedades neurodegenerativas causadas por la pérdida de las motoneuronas de la asta anterior que se caracterizan por una debilidad y atrofia muscular progresivas, provocando una gran discapacidad física, cuyas complicaciones pueden llegar a la muerte prematura, particularmente en los casos de inicio precoz [1].

La atrofia muscular espinal (AME) 5q o AME-SMN ligada al gen de supervivencia de la motoneurona 1 (*SMN1*) es la presentación más común de esta enfermedad, alcanzando el 96% de los casos [2], con una incidencia de 1:6000 a 1:10.000 nacidos vivos y una prevalencia de 1 a 2 de cada 100.000 habitantes [1]. Se estima pues, que en España hay de 800 a 1000 personas afectadas [3].

La AME está causada por una disminución de la producción de proteína SMN debida a una alteración o ausencia en el gen *SMN1* [4]. En humanos, existen varias copias del gen homólogo *SMN2*, que difiere del *SMN1* en un pequeño número de nucleótidos, que da lugar a bajos niveles de proteína SMN funcional que no son suficientes para compensar la ausencia del gen *SMN1* [5].

Aunque la AME presenta un espectro continuo de manifestaciones, existe una clasificación clásica como se muestra en la Tabla 1 (Ref. [6, 7, 8]), que considera el máximo nivel motor alcanzado sin administración de terapias modificadoras de la enfermedad (TME) y la edad de inicio de los síntomas, abarcando desde recién nacidos gravemente afectados con hipotonía grave y malformaciones congénitas (AME tipo 0), hasta inicio de la sintomatología en la edad adulta (AME tipo 4). Cabe destacar que no existe una definición homogénea para la AME tipo 4, pues se describe en literatura como de inicio progresivo en la edad adulta después de la segunda o tercer década de la vida en personas que, en general, mantienen la deambulación independiente [9, 10]. Existe también una clasificación funcional complementaria que contempla el nivel motor actual del paciente (no sedestación, sedestación, marcha) [6]. El número de copias del gen *SMN2* es un buen predictor, aunque no absoluto, del pronóstico y la evolución de la enfermedad [7, 8, 11, 12].

**Tabla 1.** **Clasificación clásica de la atrofia muscular espinal [6, 7, 8]**.

Tipo de AME	Edad inicio manifestaciones	Incidencia (%)	Máximo nivel motor alcanzado sin tratamiento específico para AME	Pronóstico de vida sin tratamiento específico para AME	Número de copias *SMN2*	Comorbilidades
0	Prenatal (disminución de los movimientos fetales)	<1%	No sedestación. Nunca alcanzan la capacidad de respirar o de alimentarse de manera independiente	<2 meses	1 copia	∙ Cardiopatía
						∙ Fracaso respiratorio
						∙ Artrogriposis
						∙ Disfagia
1	<6 meses	50–60%	No sedestación	6 meses (80%)	2 copias: 73%	∙ Compromiso respiratorio, tos inefectiva, respiración paroxística
					3 copias: 20%	∙ Debilidad muscular generalizada, contracturas, hipotonía,
					1 copia: 7%	∙ Disfagia
2	6–18 meses	30%	Sedestación, no marcha	Mediana >20 años	3 copias: 78%	∙ Insuficiencia respiratoria, tos inefectiva
					2 copias: 16%	∙ Contracturas, debilidad
					4 copias: 5%	∙ Escoliosis
3	>18 meses	10%	Marcha	Edad Adulta	3 copias: 49%	∙ Contracturas, debilidad muscular variable
					4 copias: 44%	∙ Escoliosis
					2 copias: 5%	
4	>10–20 años;	<1%	Marcha	Normal	4 copias	∙ Debilidad muscular
	>30 años					

AME, atrofia muscular espinal; SMN, survival motor neuron.

En la última década la AME ha experimentado un aumento en la supervivencia gracias a un manejo más proactivo como la introducción temprana de ventilación no invasiva y la alimentación por gastrostomía [6, 13]. Desde su aparición, los tratamientos modificadores de la enfermedad, cuyo objetivo es aumentar la producción de proteína SMN, han contribuido también de manera fundamental a este cambio significativo, con mejoras en la supervivencia en los fenotipos más graves y mejoras en los hitos motores y la calidad de vida en todos los tipos y edades [14, 15, 16]. Además, nuevas moléculas con distintas dianas terapéuticas están actualmente en desarrollo clínico (inhibidores de la miostatina o estabilizadores de la transmisión neuromuscular), con el objetivo de complementar los tratamientos modificadores actualmente comercializados y dar respuesta a las necesidades no cubiertas que a fecha de hoy sigue teniendo el colectivo AME [17, 18, 19, 20].

Existen tres tratamientos farmacológicos aprobados para uso humano actualmente centrados en aumentar la producción de proteína SMN completa, bien sea favoreciendo la inclusión del exón 7 en *SMN2* (nusinersén y risdiplam), o sustituyendo el gen *SMN1* ausente o defectuoso (onasemnogene abeparvovec-xioi) [14, 15, 16]. Su financiación en el sistema sanitario público ha ido acompañada de protocolos farmacoclínicos que en los primeros años se definieron con unos criterios de beneficio clínico más exigentes que los propios ensayos clínicos que determinaron la aprobación de las moléculas [21, 22, 23], lo que restringió el acceso y puso en riesgo la continuidad de los tratamientos en ciertos pacientes. Se presenta un resumen de los distintos tratamientos modificadores de la enfermedad comercializados en España en la Tabla 2 (Ref. [14, 15, 16, 21, 22, 23]).

**Tabla 2.** **Tratamientos modificadores de la enfermedad autorizados en España**.

Principio activo	Aprobación	Aprobación	Autorización	Posología y	Vías de acceso al tratamiento
(nombre comercial)	FDA (USA)	EMA (Europa)	España	Vía de administración	en España
Nusinersén	Diciembre 2016	Mayo 2017 [14]	Marzo 2018 [22, 23]	Vía Intratecal	∙ 2015–2017: Ensayo clínico
(Spinraza)	Indicación:	Indicación:	Indicación:	4 dosis de carga (en 2 meses) y después 1 dosis cada 4 meses	∙ Diciembre 2016 (hasta comercialización): Programa de uso en condiciones especiales
	todo AME	todo AME	con restricciones (protocolo farmacoclínico)		∙ Comercializado
Onasemnogene abeparvovec-xioi	Mayo 2019	Junio 2020 [15]	Diciembre 2021 [21]	Vía intravenosa	∙ Ensayo internacional
(Zolgensma)	Indicación:	Indicación:	Indicación:	Dosis única	∙ Marzo 2022: FoC: Programa “Free of Charge”
	AME	todos AME tipo 1; resto de tipos si ≤3 copias SMN2 y peso ≤21 kg	con restricciones (protocolo farmacoclínico)		∙ Comercializado
	<2 años de edad				
Risdiplam	Agosto 2020	Marzo 2021 [16]	Diciembre 2022 [22]	Vía Oral	∙ 2020: Ensayo clínico
(Evrysdi)	Indicación:	Indicación:	Indicación:	Dosis diaria	∙ Agosto 2020 (hasta comercialización): Programa de uso en condiciones especiales
	todo AME	AME 1 (mayores de 2 meses), 2 y 3 con ≤4 copias SMN2	con restricciones (protocolo farmacoclínico)		∙ Comercializado

FDA, Food and Drug Administration; EMA, European Medicines Agency.

Ante la necesidad de obtener un mejor conocimiento de la patología, la Organización nacional de pacientes con Atrofia Muscular Espinal (FundAME) creó en el 2015 RegistrAME “Registro Nacional de Pacientes de AME” para la recogida de datos de todo tipo de pacientes con AME en España, cuyos datos generales han sido publicados recientemente [24].

El objetivo del presente trabajo es realizar un análisis transversal, descriptivo y exploratorio de las distintas variables de interés vinculadas al estado de salud y de acceso a tratamiento de los pacientes pertenecientes a RegistrAME con AME confirmada genéticamente, con especial enfoque de los pacientes adultos.

## 2. Sujetos y Métodos

RegistrAME es una base de datos online, longitudinal, prospectiva, a largo plazo, con la participación voluntaria de todo tipo de pacientes con AME en España. El objetivo es el de recoger datos clínicos y resultados comunicados por el paciente (patient-reported outcomes, PROS) previamente definidos como relevantes para el colectivo AME [25, 26].

Para el presente trabajo se incluyeron participantes con diagnóstico de AME por ausencia o alteración de ambos alelos del gen *SMN1* a través de un informe genético confirmatorio y documentado, residentes en España en el momento del análisis, que hubieran mostrado conformidad para su participación y con expedientes de datos clínicos validados por médicos especialistas en la enfermedad que ejercieron como curadores del registro. Todos los datos incluidos en RegistrAME fueron introducidos por los pacientes o por sus cuidadores y se solicitó a los pacientes actualizar los resultados comunicados por el paciente y sus propios datos clínicos disponibles cada seis meses. Solamente los datos validados por los curadores fueron considerados para el presente estudio.

La base de datos RegistrAME es compatible con el conjunto de datos propuesto por la alianza TREAT-NMD (Global Neuromuscular Network) y cumple con los estándares éticos exigibles, ya que se realiza siguiendo la Declaración de Helsinki y la normativa legal aplicable. Todos los pacientes o sus representantes legales firmaron un consentimiento informado antes de introducir los datos en el proyecto. Además, el presente estudio cuenta con la aprobación del Comité de ética de Investigación con medicamentos (CEIm) del Hospital Universitario 12 de Octubre (aprobación 20/413).

Para la realización de este análisis descriptivo se usaron frecuencias y rangos. La realización del análisis se llevó a cabo con el programa Microsoft® Excel® 2021 MSO v.2501/Microsoft Office Professional Plus (Microsoft Corporation, Redmond, WA, USA).

## 3. Resultados

Se analizaron los datos recogidos de RegistrAME hasta junio de 2024. De los 383 pacientes registrados inicialmente, 336 cumplían con los criterios de inclusión y fueron incorporados en el análisis.

### 3.1 Características Clínicas

En las Tablas 3,4 se muestran las características clínicas de la población. A efectos del análisis, se consideraron adultos aquellos pacientes con 16 años o más, correspondiendo al 51,8% de la muestra analizada, con una distribución del 95% en tipos 2 y 3, que son formas de inicio en la infancia. Solamente un 1,7% (n = 3) presentaron la forma de inicio en la edad adulta o tipo 4. En relación al nivel de funcionalidad, un 24,7% de la población adulta indicaron no tener función útil en las manos o no poder alcanzar la boca con ellas, y el 73,6% apuntaron precisar ayuda para girar. En cambio, en el subgrupo de los menores de 16 años, estas mismas limitaciones se observaron en un 5,6% y en un 31% de la muestra respectivamente. Para la población con capacidad de mantenerse sentados (sitter), el 84% de los adultos, así como el 58,1% de los niños no eran capaces de alcanzar la sedestación de manera independiente, requiriendo ayuda para lograrlo. Finalmente, en la población ambulante, el 53,1% de la población total y el 39,4% de los adultos refirió precisar una silla de ruedas para desplazarse.

**Tabla 3.** **Características clínicas distribuidas por grupos etarios**.

Variable		Total		<16 años		≥16 años	
		n	%	n	%	n	%
Sujetos incluidos		336	100%	162	48,2%	174	51,8%
Edad media (años)				7,5		34	
Edad (rango)		(5 m–79 a)		(5 m–15 a)		(16–79 a)	
Sexo							
	Masculino	186	55,4%	88	54,3%	98	56,3%
Tipo de AME							
	Tipo 1	78	23,2%	71	43,8%	7	4%
	Tipo 2	157	47%	71	43,8%	86	49,4%
	Tipo 3	98	29,2%	20	12,3%	78	44,8%
	Tipo 4	3	0,9%			3	1,7%
Copias *SMN2*							
	2 copias	81	24,1%	69	42,6%	12	6,9%
	3 copias	191	56,8%	81	50%	110	63,2%
	4 copias	37	11%	6	3,7%	31	17,8%
	Desconocido^*^	27	8%	6	3,7%	21	12,1%
Estatus motor							
	Sin sedestación (Non-sitter)	86	25,6%	26	16%	60	34,5%
	Sedestación independiente (Sitter)^†^	186	55,4%	105	64,8%	81	46,6%
	Ambulante (Walker)^‡^	64	19%	31	19,1%	33	19%
Función miembros superiores							
	No tengo función útil con las manos	16	4,8%	5	3,1%	11	6,3%
	No puedo llevar las manos hasta la boca, pero tengo función motora útil en las manos	36	10,7%	4	2,5%	32	18,4%
	Puedo llevar las manos hasta la boca	110	32,7%	32	19,8%	78	44,8%
	Puedo subir los brazos por encima de la cabeza	174	51,8%	121	74,7%	53	30,5%
Giro							
	No puedo girar	112	33,3%	21	13%	91	52,3%
	Solo puedo girar parcialmente	66	19,6%	29	17,9%	37	21,3%
	Puedo girar completamente	158	47%	112	69,1%	46	26,4%
Cirugía de escoliosis							
	Intervención	125	37,2%	26	16%	99	56,9%
Tratamiento TME							
	En tratamiento en la actualidad	298	88,7%	162^§^	100%	136^§^	78,2%

TME, tratamiento modificador de la enfermedad. ^*^El informe genético aportado no contiene el número de copias. ^†^Se considera sedestación independiente si el individuo logra mantenerse sentado de manera independiente, independientemente que logre sentarse por su cuenta. ^‡^Se define ambulante aquel que camina al menos 10 metros sin asistencia de dispositivos o de terceros. ^§^Algunos individuos actualmente tratados iniciaron su tratamiento en programas de uso en condiciones especiales (28 pacientes <16 años y 13 pacientes ≥16 años).

**Tabla 4.** **Características clínicas específicas según estado motor**.

Variable		Total		<16 años		≥16 años	
		n	%	n	%	n	%
Sedestación		186		105		81	
	No soy capaz de sentarme solo, pero puedo mantenerme sentado de forma independiente	129	69,4%	61	58,1%	68	84%
	Soy capaz de sentarme solo y mantenerme de pie	17	9,1%	12	11,4%	5	6,2%
	Soy capaz de sentarme solo y no me mantengo de pie	31	16,7%	26	24,8%	5	6,2%
	Doy algunos pasos. No alcanzo una distancia de 10 metros de manera independiente	9	4,8%	6	5,7%	3	3,7%
Ambulación		64		31		33	
	Camino de forma independiente, nunca uso silla de ruedas	30	46,9%	10	32,3%	20	60,6%
	Camino solo en casa	10	15,6%	8	25,8%	2	6,1%
	Camino tramos cortos, para tramos largo uso silla de rueda	24	37,5%	13	41,9%	11	33,3%

**Fig. 1.**
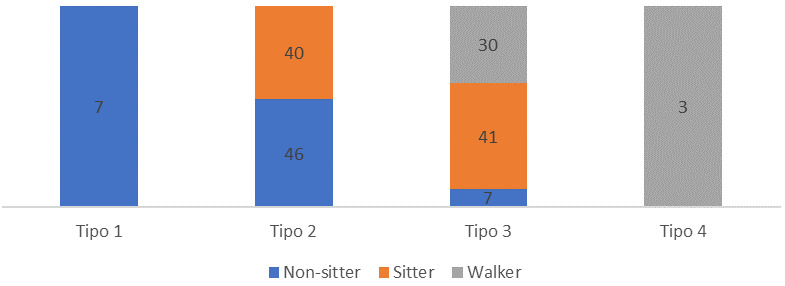
**Distribución de la subpoblación adulta según tipo de atrofia muscular espinal y estado funcional**.

En la Fig. 1 se muestran los resultados obtenidos al cruzar las clasificaciones clásica y funcional en la subpoblación de los sujetos adultos. Así, el 52% de los adultos tipo 2 perdieron la capacidad de sentarse sin apoyo, convirtiéndose en non-sitters. El 62% de los adultos tipo 3 experimentaron la pérdida de la capacidad de caminar (sitters), o dificultades para mantenerse sentados sin apoyo (non-sitters).

### 3.2 Acceso a Tratamiento

El 88.7% de la población analizada estaba recibiendo un TME en el momento del corte para realizar el presente análisis. Sin embargo, la distribución por grupos de edad refleja que el 100% de los niños estaban en tratamiento, mientras que el porcentaje en la población adulta fue de 78,2% (Tabla 3). Para esta población, la edad media de inicio de tratamiento de las personas con tipo 1 fue 17,7 años (9–24), para el tipo 2 fue 25,5 años (10–65) y para el tipo 3 fue de 35,5 años (10–74).

Así, la población no tratada correspondió al 21,8% (n = 38), todos ellos del grupo adulto. Este grupo incluyó dos situaciones en función de si recibieron TME previo. Con respecto a la población sin tratamiento activo en el momento del análisis, nueve sujetos habían recibido medicación y se discontinuó ya sea por efectos adversos descritos en ficha técnica (n = 2), bien por cumplimiento de las condiciones del protocolo farmacoclínico de nusinersén (n = 6), o por ambos criterios (n = 1). Respecto a la población que nunca recibió tratamiento (n = 29), correspondió al 17.4% (n = 15) de los AME tipo 2, al 14.1% (n = 11) de los AME tipo 3 y al 100% (n = 3) de los AME tipo 4, con una edad media de 39.8 años (18–78 años). Los motivos para descartar el tratamiento se agruparon en: causas anatómicas (n = 4), decisión del paciente (n = 7), valoración del médico (n = 1), a la espera de tratamiento (n = 10) y desconocido (n = 7).

**Fig. 2.**
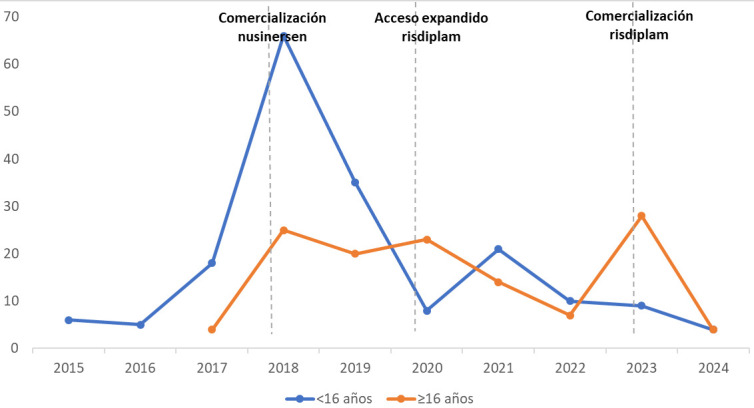
**Cantidad de nuevos inicios de tratamiento modificador de la atrofia muscular espinal según grupos de edad**.

**Fig. 3.**
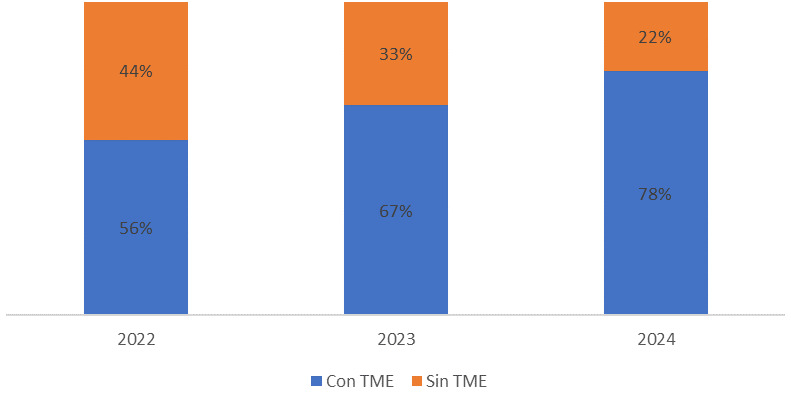
**Evolución del porcentaje de adultos con atrofia muscular espinal según estado de tratamiento**.

En referencia al número de pacientes tratados por cualquier vía desde los incluidos en ensayos clínicos en 2015 hasta la actualidad, correspondiendo al período en el que ha habido tratamientos farmacológicos disponibles, en la Fig. 2 se muestra la evolución en la cantidad de inicios de tratamiento por año de ambas subpoblaciones y en la Fig. 3 se representa la evolución del porcentaje de los adultos en función de su estado de tratamiento.

Un 18,5% de la muestra (n = 62) participaron o están actualmente en alguno de los ensayos clínicos para el tratamiento de la AME, incluyendo terapias dependientes de SMN, inhibidores de la miostatina y otros. La mayoría de la población en ensayo fueron incluidos en edad pediátrica y solamente 11 lo hicieron ya en edad adulta. Cabe destacar que cinco pacientes reclutados en edad pediátrica tuvieron la opción de participar en dos ensayos de tratamientos diferentes consecutivamente.

## 4. Discusión

El presente estudio muestra los datos de registro de una gran cohorte de sujetos con AME y permite valorar las diferentes condiciones de acceso a tratamientos y el distinto impacto de la enfermedad en los pacientes adultos, así como ofrecernos una perspectiva de la evolución de la enfermedad.

El diagnóstico de AME tipo 2 es la forma predominante en la población total (47%). Mientras que en la subpoblación pediátrica predominan los tipos 1 (43,8%) y tipo 2 (43,8%), en la subpoblación adulta las formas predominantes son los pacientes tipo 2 (49,4%) y tipo 3 (44,8%). A pesar del bajo porcentaje de pacientes con el subtipo más grave o tipo 1 en la subpoblación adulta (1%), nótese que es en la edad adulta cuando observamos mayor discapacidad en esta serie. Esto que puede ser el reflejo del deterioro físico producto de la progresión de la enfermedad que experimentaron los adultos tipo 2 y tipo 3 en la historia natural de la enfermedad previa a la aparición de los tratamientos modificadores.

A pesar de la baja incidencia conocida de AME tipo 4 menor al 1%, su prevalencia en otras cohortes puede alcanzar aproximadamente el 5% de la población con AME [12], lo que se explica principalmente por su baja tasa de mortalidad comparada con otros tipos de AME y comparable a la población general, lo que contribuye a una acumulación progresiva de casos a lo largo del tiempo. Así pues, resulta llamativa la baja prevalencia observada en este análisis (<1%), aunque es similar a otras cohortes presentadas [27, 28, 29]. Esta discrepancia en la literatura podría atribuirse, en parte, a la falta de consenso sobre la definición del tipo 4 dependiendo de la clasificación a la que se haga referencia [12, 30, 31]. Además, podría deberse también a su frecuente asimilación a los AME tipo 3, dado que clasificaciones anteriores solo incluían los tipos 1, 2 y 3. Asimismo, es posible que el infra diagnóstico de este subtipo contribuya a la menor prevalencia reportada. Seguramente, todos los factores mencionados contribuyen a que la historia natural de los pacientes tipo 4 no sea bien conocida y podamos encontrar en la bibliografía fenotipos muy diversos de pacientes clasificados como AME tipo 4, desde pacientes que no se sientan hasta individuos ambulantes [32]. Por todo ello, sería conveniente trabajar en una definición homogénea para diagnosticar a los pacientes de tipo 4 y determinar adecuadamente el impacto de la enfermedad y la discapacidad asociada, así como las necesidades no cubiertas de este subgrupo. Esto es especialmente relevante en nuestro entorno, puesto que este tipo de paciente tiene limitado en la actualidad el uso de tratamientos modificadores de la enfermedad debido a las restricciones marcadas por el protocolo farmacoclínico vigente en este momento. Todo esto sugiere que, al igual que en otros estudios, solamente el tipo no es un criterio adecuado o suficiente para evaluar la gravedad y el impacto de la enfermedad, y que existe la necesidad de avanzar en un consenso para una clasificación que refleje el estado real del individuo y su grado de discapacidad [32].

Las formas más comunes en la edad adulta se originan en la infancia (tipos 2 y 3), por lo que la gran mayoría de los adultos de la presente serie acumulan muchos años de evolución de enfermedad, lo que se traduce en una mayor limitación funcional y pérdida de autonomía. Según nuestro estudio, cerca del 25% de los adultos no pueden alimentarse de manera independiente, al no tener función útil en las manos o no poder alcanzar la boca con sus manos. Además, la mayoría de los adultos requieren asistencia durante la noche para el posicionamiento al no tener capacidad de girar sin ayuda, convirtiéndolos en grandes dependientes. Otro aspecto a considerar son los porcentajes de personas de la muestra que, aunque consiguen sentarse, no lo logran por sí mismos, necesitando ayuda de otra persona (84% adultos y 58,1% niños). Tampoco los individuos que deambulan (incluyendo los pacientes tipo 4) están exentos de presentar algún tipo o grado de discapacidad puesto que, en algunos casos refieren no poder subir escaleras, tener caídas frecuentes (con la consiguiente fractura que puede devenir), o no poder levantarse del suelo o una silla, imposibilitándolos en la realización de tareas del día a día de manera independiente, como usar el transporte público, por ejemplo [33]. Cabe destacar que aproximadamente el 40% de los adultos que deambulan en nuestra serie caminan distancias cortas en espacios interiores, precisando, generalmente, silla de ruedas para sus desplazamientos fuera del hogar. Todo ello nos conduce a reflexionar sobre la necesidad de valorar la calidad del hito motor alcanzado y el impacto que produce la enfermedad en la autonomía o la vulnerabilidad del propio sujeto. En este ámbito, la incorporación de los PROS específicos para la AME constituyen una herramienta de gran utilidad y por este motivo varios estudios para la determinación de PROS específicos para la AME están actualmente en desarrollo [26, 34].

Acerca del acceso a los tratamientos, es remarcable que la totalidad de población menor de 16 años se encuentra en tratamiento, mientras que el 78% de la población adulta del registro está en tratamiento modificador de la enfermedad en este corte de datos. Aunque la aparición de la terapia oral risdiplam, ha permitido que un mayor porcentaje de población adulta con AME tenga acceso a tratamiento, aproximadamente uno de cada cinco adultos sigue sin acceder a tratamiento. Algunos de los motivos por los que actualmente hay personas con AME que no reciben tratamiento incluyen: una falta de actualización acerca del estado del tratamiento actual de la enfermedad, dificultades técnicas en la administración debido al reto anatómico secundario a las deformidades o la cirugía de columna, la propia decisión del paciente (vinculada al rechazo de la vía intratecal para ser tratado) o estar a la espera de recibir un tratamiento. Con la decisión positiva de financiación para nusinersén en 2018 y risdiplam oral desde diciembre del 2022, la mayoría de estos motivos no deberían ser una limitación para el tratamiento e invitan a la reflexión acerca de la necesidad de agilizar los procesos de información y acceso de esta población a los tratamientos actuales. Con respecto a los motivos de discontinuación, el protocolo farmacoclínico inicial para nusinersén estipuló unos criterios de iniciación y retirada del tratamiento que impactaron en la continuidad del fármaco en numerosos casos, con independencia del tipo de AME y de la edad del paciente, llevando a la comunidad médica a manifestar la necesidad urgente de actualizar el protocolo [35]. La retirada del fármaco en esta enfermedad de carácter degenerativo pudo suponer una pérdida irreversible de función motora en algunos casos, lo que pudo acompañarse de sufrimiento y disconformidad por parte tanto de algunos pacientes como de sus cuidadores.

Los resultados reflejan que el 18,5% del total de la población del estudio ha tenido acceso a alguno de los tratamientos farmacológicos (terapias *SMN*, inhibidores de miostatina u otros) gracias a su inclusión en los programas de desarrollo clínico previos a la aprobación de los mismos e incluso se han beneficiado de una combinación secuencial de tratamientos [36]. Por otro lado, el volumen de participantes incluidos en programas de uso en condiciones especiales refleja que constituyen una herramienta de gran valor para la comunidad AME ya que permitieron el tratamiento temprano en ambas subpoblaciones y sin coste para el sistema de salud. Además, la puesta en marcha de estos programas hizo posible que haya pacientes en tratamiento que no podrían haberse tratado en las condiciones posteriormente autorizadas (pacientes traqueotomizados, por ejemplo) puesto que se utilizaron criterios de inclusión más flexibles que en las condiciones de autorización [37, 38].

Así pues, a pesar del evidente impacto de la enfermedad en la población adulta con AME y de la disponibilidad de dos tratamientos para ellos, persiste una dificultad importante en el acceso a estos tratamientos. Podría existir una infravaloración de la situación clínica que produce la pérdida de autonomía en esta población que puede explicar la falta de equidad en el acceso al tratamiento. Teniendo en cuenta la importancia de la autonomía de la población con AME y la independencia de sus cuidadores, el mantenimiento de la función motora o funciones como el girarse en la cama sin ayuda, la destreza digital o conseguir sentarse de forma autónoma tiene enormes implicaciones para este colectivo. Aspectos como la disminución de la fatigabilidad, una correcta deglución o mantener el tono de voz para comunicarse, juntamente con la preservación de su autonomía, son vitales para el paciente y no han sido suficientemente contemplados en los programas de investigación para el desarrollo clínico de las moléculas autorizadas como tampoco en el protocolo farmacoclínico.

### Limitaciones del Estudio

Atendiendo a los datos epidemiológicos de personas con AME en España, la muestra presentada y su diversidad contribuyen a la representatividad de la misma. Además, la validación y depuración de los datos introducidos por los pacientes por parte de un experto clínico les confiere robustez y fiabilidad. Aun así, este estudio no está exento de limitaciones, como las limitaciones propias de un estudio de vida real con datos registrados por el propio sujeto participante, por lo que se debe tener en cuenta que los datos de información clínica previos al alta del paciente en el registro se introducen de forma retrospectiva. La representabilidad de los resultados obtenidos se ve circunscrita a personas con la enfermedad que pertenezcan a la asociación, lo que puede producir ciertos sesgos en relación a la distribución territorial desigual de los participantes, o una menor participación de individuos que no consideren útil este tipo de herramientas para el incremento del conocimiento de la enfermedad, o con un elevado grado de aislamiento. Juntamente con la imposibilidad de participar de aquellos sujetos aún no diagnosticados, todos estos factores han podido afectar a la variabilidad y el perfil de pacientes incluidos en este estudio.

## 5. Conclusiones

A la par de la importancia del éxito de los tratamientos modificadores de la enfermedad y la necesidad de la equidad en el acceso, es relevante mantener el enfoque multidisciplinar y el valor de los cuidados estandarizados, pues han demostrado cambiar el paradigma de la enfermedad. Los resultados de este estudio muestran la evolución del acceso a los tratamientos modificadores de la población adulta con AME y reflejan la necesidad de seguir mejorando el acceso a los mismos, pues sigue habiendo un porcentaje de pacientes sin tratamiento. Los resultados presentados constatan un perfil de un individuo adulto con AME que tendría menor autonomía e independencia que el colectivo pediátrico, con necesidades sin cubrir que no han sido identificadas en los programas de investigación de los tratamientos disponibles llevados a cabo hasta la fecha. Así, son necesarias investigaciones que nos ayuden a identificar las necesidades existentes a fecha de hoy en los pacientes adultos con AME y diseñar ensayos específicos para que este colectivo pueda acceder a la investigación y desarrollo de los fármacos que se estudien para esta enfermedad y así garantizar el acceso a los mismos. Para un mejor conocimiento de la enfermedad y de las necesidades no cubiertas, el registro presentado constituye una herramienta útil que contribuye a visibilizar, identificar y seguir la evolución de las personas con AME.

## Disponibilidad de Datos y Materiales

Los conjuntos de datos utilizados y/o analizados durante el presente estudio están disponibles a pedido razonable del autor correspondiente.

## Contribuciones de los Autores

MDL, MD y ET: Conceptualización, diseño, interpretación de los datos y revisión sustancial del manuscrito. MGC: Conceptualización, diseño, adquisición, análisis e interpretación de los datos y revisión sustancial del manuscrito. Todos los autores leyeron y aprobaron el manuscrito final. Todos los autores han participado lo suficiente en el trabajo y han acordado ser responsables de todos los aspectos del mismo.

## Aprobación Ética y Consentimiento Informado

La base de datos RegistrAME es compatible con el conjunto de datos propuesto por TREAT-NMD y cumple con los estándares éticos exigibles, ya que se realiza siguiendo la Declaración de Helsinki y la normativa legal aplicable. Todos los pacientes o sus representantes legales firmaron un consentimiento informado antes de introducir los datos en el proyecto. Además, el presente estudio cuenta con la aprobación del CEIm del Hospital Universitario 12 de Octubre (aprobación 20/413).

## Agradecimientos

FundAME agradece a todos los pacientes y sus familias que contribuyen al Registro. Agradecemos a Cristina Puig su asistencia en la redacción y envío del manuscrito.

## Financiación

Este trabajo ha sido sufragado en su totalidad por FundAME y no se han recibido ayudas externas derivadas del sector comercial, el sector público o entidades sin ánimo de lucro.

## Conflicto de Intereses

Maria Grazia Cattinari ha recibido ayuda de Roche para la asistencia a congresos científicos. Mencía de Lemus ha realizado tareas de asesoramiento para Roche. Maria Dumont declara no tener conflictos de interés. Eduardo Tizzano ha recibido financiación para estudios de investigación de parte de Ionis/Biogen y Roche y por tareas de asesoramiento para Avexis, Novartis, Biogen, Roche, Cytokinetics, Biologix y PTC.
